# DOPNet: Achieving Accurate and Efficient Point Cloud Registration Based on Deep Learning and Multi-Level Features

**DOI:** 10.3390/s22218217

**Published:** 2022-10-27

**Authors:** Rongbin Yi, Jinlong Li, Lin Luo, Yu Zhang, Xiaorong Gao, Jianqiang Guo

**Affiliations:** School of Physical Science and Technology, Southwest Jiaotong University, Chengdu 622731, China

**Keywords:** registration, deep learning, point clouds, attention

## Abstract

Point cloud registration aims to find a rigid spatial transformation to align two given point clouds; it is widely deployed in many areas of computer vision, such as target detection, 3D localization, and so on. In order to achieve the desired results, registration error, robustness, and efficiency should be comprehensively considered. We propose a deep learning-based point cloud registration method, called DOPNet. DOPNet extracts global features of point clouds with a dynamic graph convolutional neural network (DGCNN) and cascading offset-attention modules, and the transformation is predicted by a multilayer perceptron (MLP). To enhance the information interaction between the two branches, the feature interaction module is inserted into the feature extraction pipeline to implement early data association. We compared DOPNet with the traditional method of using the iterative closest point (ICP) algorithm along with four learning-based registration methods on the Modelnet40 data set. In the experiments, the source and target point clouds were generated by sampling the original point cloud twice independently; we also conducted additional experiments with asymmetric objects. Further evaluation experiments were conducted with point cloud models from Stanford University. The results demonstrated that our DOPNet method outperforms these comparative methods in general, achieving more accurate and efficient point cloud registration.

## 1. Introduction

3D point clouds contain abundant spatial information. This kind of 3D data is significant in many areas, such as 3D target detection [1], 3D localization [2], and robotics [3]. Position mismatching is inevitable due to the inconsistency of acquisition angles and reference frames during the data acquisition process [4]. Therefore, point cloud registration is a fundamental task of point cloud data processing, and it is crucial for point cloud data applications to achieve highly accurate registration.

Current registration methods can be generally divided into two branches: traditional methods and learning-based ones. The classic traditional method is the iterative closest point (ICP) algorithm [5], which is simple in theory and can achieve suitable registration accuracy with a small initial spatial difference. However, ICP has non-negligible limitations, such as its sensitivity to the initial position of the point clouds, frequently falling into local minima, disappointing efficiency, and so on [6]. Researchers have successively proposed Go-ICP [7], normal iterative closest point (NICP) [8], generalized ICP (GICP) [9], and many other ICP-style algorithms to solve these problems, which do perform better than ICP, but these variants still fail to eliminate the inherent defects of ICP.

Benefiting from parameter-learning ability, learning-based registration algorithms are generally more efficient and effective than traditional algorithms. PointNet [10] and its improved version PointNet++ [11] can directly analyze point cloud data. PCRNet [12] constructs an iterative registration network based on PointNet, with simple structural complexity and satisfactory registration error. PointNetLK [13] embeds the point clouds into a higher-dimensional space using PointNet, while adopting the improved Lucas–Kanade algorithm [14] instead of the common T-net-style network in PointNet to align the point clouds. Dynamic graph convolutional neural network (DGCNN) [15] designs the EdgeConv module to extract local features. Deep closest point (DCP) [16] combines DGCNN with Transformer [17] to generate soft registration, and then approximates the ground truth by iterating. It has achieved significant improvement compared with previous registration algorithms.

However, these algorithms have various defects in feature extraction, calculation efficiency, or registration accuracy, most of which are generally sensitive to noise. Considering these factors, we propose a learning-based end-to-end point cloud registration method: DOPNet. DOPNet mainly consists of two parts: the feature extraction layer and the transformation prediction layer. The feature extraction layer is made up of three parts: (1) Using DGCNN to extract initial features and embed the point clouds into a higher-dimensional space; (2) cascaded offset-attention modules are utilized [18] for further feature extraction; (3) the feature interaction module is inserted to optimize the registration performance. In the transformation prediction layer, seven parameters indicating the spatial transformation are predicted by a multilayer perceptron (MLP). For some methods, experiments were conducted with two identical point clouds (e.g., DCP). We obtained the source and target point clouds by sampling the original point cloud twice independently to improve the precision. 

The main contributions of this paper can be summarized as follows:

1. We propose an end-to-end registration network that can efficiently extract multiple features of point clouds using DGCNN and an attention mechanism. A feature interaction module is inserted into the feature extraction pipeline to achieve high-quality registration.
2. The source and target point clouds were generated by independent sampling, and additional experiments on asymmetric objects from Modelnet40 were conducted, which to some degree achieved a more rational setting for the registration experiments.
3. The proposed method was compared with five other registration methods. Adequate experiments verify that DOPNet is capable of extracting more salient hybrid information, as it achieves better registration accuracy and robustness than other methods.

## 2. Related Work

### 2.1. Traditional Methods

ICP [5] and its variants are representative methods of traditional registration algorithms. ICP constructs a rigid transformation matrix by searching the nearest point sets between two point clouds, and then iterating until the accuracy reaches the threshold. ICP is an effective optimal registration method without sophisticated procedures like segmentation or feature extraction. However, the iterative computation and the process of searching the nearest point sets still cause extensive computation expenditure, and it is prone to local minima and sensitive to initial positions, as ICP only considers point-to-point distances. Generalized ICP (GICP) [9] combines point-to-point, point-to-plane, and plane-to-plane strategies to improve registration accuracy and robustness. Normal iterative closest point (NICP) [8] takes into consideration the normal vectors and the local curvature, and thus it enhances the utilization of local structure information. Variants of ICP do make improvements to some extent, but there are still limitations in terms of calculation efficiency, accuracy, and robustness. 

### 2.2. Learning-Based Methods

Learning-based methods tend to possess better robustness and generalization than traditional ones because of their parameter-learning ability. PointNet [10] is the first neural network which can directly process 3D point clouds; it utilizes an MLP to achieve feature mapping without additional transformation. Its improved version, PointNet++ 11, enhances the utilization of local features with extensive computation cost because of its iterative feature extraction. Since then, various methods for processing point cloud data have been proposed, such as DGCNN [15], PointCNN [19], FCGF [20], and so on.

In terms of point cloud registration, PointNetLK [13] leverages the Lucas–Kanade (LK) algorithm, which is generally applied in image registration to solve the point cloud registration challenge and successfully improves registration accuracy, but its iterative computation style renders the calculation efficiency inferior. CorsNet [21] extracts global features using PointNet, in which the high-dimensional features are concatenated with the 64-dimensional features extracted by PointNet to enhance the utilization of local features. However, such stitching is not rigorous due to PointNet’s inherent deficiency in extracting local features [11]. DeepGMR [22] constructs a registration network based on the Gaussian mixture model (GMM), and it formulates registration as the process of minimizing the KL divergence [23] between two probability distributions modeled as mixtures of Gaussians. The 3DRegNet [24] network utilizes pointwise correspondence to align two point clouds. A key point detector in DeepVCP [25] is designed to enhance registration accuracy via learning searches of qualified key point sets. FMR [26] constrains the global feature distance of the inputs with an extra decoder.

DCP [16] adopts DGCNN [15], which is more capable of extracting local features compared with PointNet, to map point clouds into the higher-dimensional space, and it approximates soft matching through transformer and pointer layers. DCP is an effective migration application of an attention mechanism from natural language processing (NLP) to point cloud registration, but it relies on special local features of point clouds to generate effective feature-point correspondences. Two identical point clouds were employed for experiments, which makes the evaluation results less rigorous.

### 2.3. Attention Mechanism

The attention mechanism is proposed in [17], which is widely applied in the NLP and computer vision (CV) fields [27]. In terms of point clouds, point cloud transformer (PCT) [18], point transformer (PT) [28], and DCP, etc. use an attention mechanism to extract features or to improve their feature-extraction ability. PREDATOR [29] applies cross-attention to construct an overlap-attention module and capture global pictures. Lepard [30] directly predicts the transformation matrix with cascaded self-attention and cross-attention modules. DeepUME [31] achieves accurate registration based on the combination of deep learning and a universal manifold embedding algorithm, while the transformer structure is leveraged to extract features.

PCT proposes offset attention (OA), which is an improvement based on self-attention. OA can be stacked in the point cloud processing pipeline to construct a multi-level feature extraction network, which can be used for different tasks such as semantic segmentation and classification of point clouds. This modified attention module is an excellent migration application of the transformer structure from NLP to point cloud data, and it is also more applicable to the data structure of point clouds, which inspired us to obtain abundant feature information with cascaded OA modules.

## 3. Problem Statement

In terms of the two given point clouds *P_S_* and *P_T_* with the same geometry and different spatial position, where $P_{s}=\{s_{1},s_{2},\ldots ,s_{n}\}\subset R^{3}$ and $P_{t}=\left\{t_{1},t_{2},\ldots ,t_{n}\right\}\subset R^{3}$ denote the source point cloud and the target point cloud, respectively, point cloud registration is aimed at finding a rigid spatial transformation *ST* that can align *P_S_* and *P_T_* by acting *ST* on *P_S_*. ST⊂SE(3) is defined as:

(1) $$ST=\left[\begin{array}{cc} R & T\\ 0 & 1 \end{array}\right]$$

Here, R⊂SO(3) and $T\subset R^{3}$ denote the rotation matrix and the translation vector, respectively, and the rotation matrix is transformed from a quaternion q=w+xi+yj+zk by Equation (2):

(2) $$R=\left[\begin{array}{ccc} 1-2y^{2}-2z^{2} & 2xy-2wz & 2xz+2wy\\ 2xy+2wz & 1-2x^{2}-2z^{2} & 2yz-2wx\\ 2xz+2wy & 2yz+2wx & 1-2x^{2}-2y^{2} \end{array}\right]$$

The translation vector is defined as Equation (3), where *t_x_*, *t_y_*, and *t_z_* are three translation parameters.

(3) $$T=\left[\begin{array}{c} t_{x}\\ t_{y}\\ t_{z} \end{array}\right]$$

Thus, the ultimate goal of our algorithm can be formulated as solving seven parameters ${\left[t_{x},t_{y},t_{z},w,x,y,z\right]}^{T}$, and then the spatial transformation can be derived.

## 4. Methodology

### 4.1. Network Architecture

**Figure 1:**
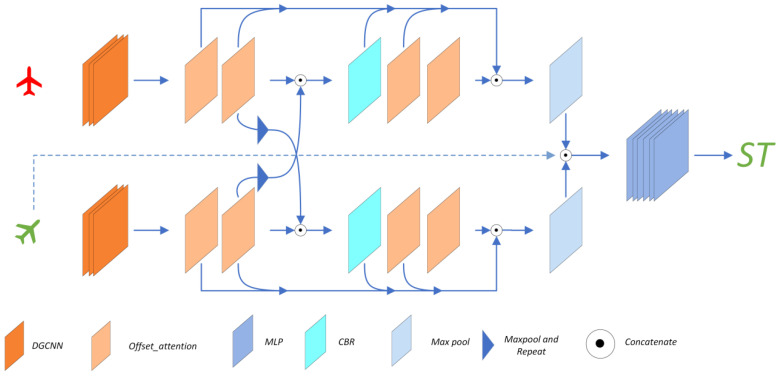
Network architecture of DOPNet, including DGCNN, cascading OA, feature interaction module, and MLP.

Figure 1 shows the structure of DOPNet. The name DOPNet denotes the DGCNN, OA, and MLP, which are the main structural parts of the network. The model is mainly composed of the feature extraction layer and the transformation prediction layer. In summary, we utilize a Siamese network that consists of DGCNN, OA modules, and feature interaction modules as the feature extraction layer. An MLP in the form of PointNet plays the role of the transformation prediction layer, which finally outputs seven parameters to derive the predicted transformation. 

### 4.2. Feature Extraction Layer

The first stage of the pipeline is embedding the original inputs *P_S_* and *P_T_* into a higher-dimensional space using DGCNN. For the *i*-th point *p_i_* of the point clouds, DGCNN constructs a local neighbor graph by searching its *k* nearest neighbors with the KNN (k-nearest neighbor) algorithm, and the graph can characterize the local geometric structure of the point clouds. Then the EdgeConv module is applied to extract corresponding edge features *F* on the edges connected between the nodes and their neighbors. The forward mechanism is formulated as:

(4) $$F=h_{\theta }\left[K_{\theta }(pi,k),\forall i\in N\right]$$

where *N* denotes the number of points of the corresponding point cloud, $K_{\theta }$ denotes the KNN algorithm, and $h_{\theta }$ denotes EdgeConv. Compared with PointNet, DGCNN is able to extract more abundant structural information from the point sets by dynamically updating the graph structure between different layers, which enables DGCNN to outperform PointNet in capturing local features. This claim will be proven in the ablation experiments in Section 5.5.

**Figure 2:**
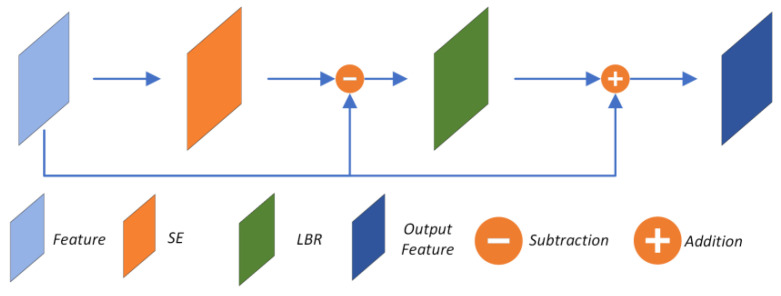
Network architecture of offset-attention, including features, self-attention, and LBR.

DGCNN can extract a wealth of geometric information, but the semantic information in the feature space is insufficient. Thus, four OA modules were employed for further multi-layer feature extraction after DGCNN to excavate more semantic feature information, and Figure 2 shows the structure of the OA module. The *l*-th OA takes the feature *F^l−^*^1^ of dimension (*N*,*d*) extracted by the previous network as input. First, the feature is re-weighted by the self-attention module without dimensional variation, then the subtraction is made between the weighted feature and the original feature. The output *F^l^* is finally generated by adding the original feature to the output of the LBR (linear + BatchNorm + ReLU) layer. Note that the OA uses softmax + *l*1Norm instead of the original scale+softmax [19] to calculate attention scores in the self-attention module; such alteration can enhance the attention weights of meaningful features and weaken the effects of noise. The mechanism is shown in Equation (5).

(5) $$F^{l}=F^{l-1}+\left[F^{l-1}-{SE}^{l}(F^{l-1})\right]$$

Here, the superscript letters denote the number of OAs. The OA is designed based on self-attention, it enhances the robustness against noise, and the utilization of the feature offsets enriches the semantic information contained in the output features, which also enhances the utilization of previous features. Its residual structure minimizes the adverse effects of training deeper neural networks, which makes it suitable for constructing a deep stacked feature extraction network. Thus, four cascaded OA modules are leveraged as the multilevel feature extraction layer to construct more comprehensive features.

In order to achieve higher registration accuracy, the global features ought to be comprehensive representations of the two point clouds’ structural and feature information. The combination of DGCNN and OA can extract satisfactory features in one branch, but the features of the two branches are uncorrelated, which means the final global features might be not applicable. Considering that case, we inserted the feature interaction module (FI) into the feature extraction pipeline to establish information connections. Such connections make the two feature extraction branches gain information from each other early on, before constructing global features to mine more desired features. Equation (6) shows the mechanism of FI for the source point cloud branch.

(6) $$F_{s}^{i}=Conv(cat\{F_{s},R\left[Max(F_{t}),N_{s}\right]\})$$

Here, *F_s_* and *F_t_* represent the low-dimensional local features of *P_S_* and *P_T_*, respectively, extracted by the previous network, and *N_s_* denotes the number of points of *P_S_*, while *R* denotes repeating the features *N_s_* times in the spatial dimension. For another branch, this is still applicable by changing the number of points and swapping the position of *F_s_* and *F_t_*. FI is inserted into the pipeline after the second OA module to make the features interact early on. Furthermore, the dimension of features will be doubled by FI, which ought to make the subsequent OA modules extract more latent features in the higher-dimensional space. 

Finally, the outputs of FI and OA are concatenated through skip connection to conduct multi-level feature extraction. The multi-level features contain multi-dimensional structural and semantic information, which comprehensively characterizes the point clouds. Then the features are processed by max pooling to obtain global features.

### 4.3. Transformation Prediction Layer

The total features are generated by stitching the global features of *P_S_* and *P_T_*, and then the total features are concatenated with the spatial coordinates of *P_T_* as the input of the transformation prediction layer, which aims at aligning the global features to the coordinates of each point of *P_T_*. Finally, the hybrid features contain multi-dimensional features and the Euclidean spatial information of *P_T_*, which further enhances the feature linkage between *P_S_* and *P_T_*. Such composite features enable the transformation prediction layer to predict more accurate transformation.

An MLP is employed as the transformation prediction layer. The parameter-learning-capability of the MLP makes it able to process the composite features more efficiently, and it can mine the complex latent information to directly predict the transformation matrix. The output of the transformation prediction layer is a 1 × 7 vector ${\left[t_{x},t_{y},t_{z},w,x,y,z\right]}^{T}$. The first three parameters denote the translation vector *T*, and the last four parameters denote the quaternion *q*. 

### 4.4. Loss Function

The goal is aligning the given point clouds by predicting accurate transformation, so the predicted transformation and the true transformation are chosen as the variables to construct the loss function, which is defined as Equation (7).

(7) $$Loss=\left\|Q_{pre}-Q_{gt}\right\|+\lambda {\left\|t_{pre}-t_{gt}\right\|}_{2}$$

where *t* and *Q* denote the translation vector and the rotation matrix, respectively, while the subscripts *pre* and *gt* denote the predicted results and the ground truth respectively. *λ* is the weight coefficient, which adjusts the model’s sensitivity to rotation and translation, it is empirically set to 1.5.

## 5. Experiments

### 5.1. Implementation Details

We used Modelnet40 [32], which covers 12,311 3D CAD models from 40 categories, and the models from the Stanford 3D Scanning Repository [33] as the experimental data sets. However, it was not reasonable for some symmetrical objects from Modelnet40 (bottle, bowl, cone, cup, flowerpot, lamp, tent, vase, etc.) to evaluate the registration accuracy by using the present metrics [34]. Thus, dual experiments were conducted on total objects (TO) and asymmetric objects (AO) by removing those symmetrical objects. We used cosine annealing to adjust the learning rate with an initial learning rate of 0.0001 and adopted the Adam optimizer [35] to optimize the network parameters for 350 epochs of training. All experiments were conducted with an AMD 5600X CPU and an NVIDIA GeForce RTX 3060 GPU. 

### 5.2. Comparison and Evaluation Metrics

DOPNet was compared with ICP [5] and four learning-based methods, including DCP [16], PointNetLK [13], DeepGMR [22], and CorsNet [21]. DOPNet was temporarily designed without considering partial-to-partial registration. Our first motivation was choosing algorithms that had similar application scenarios. In addition, those methods which were similar in construction or method of manipulating features also could be ideal choices. Thus, we chose five methods as comparisons to validate the registration ability and methodology of DOPNet. 

For all training and testing, unless otherwise specified, *P_S_* and *P_T_* were generated by randomly sampling 1024 points twice from the original point cloud; the rotation angles were randomly generated in [0°, 45°] and the initial translation distances were randomly selected in [−0.5, 0.5] as the true transformation. We measured the root mean square error (RMSE) between the predicted value and the ground truth for anisotropic error evaluation. The metrics proposed in RPMNet [36] were employed for isotropic error evaluation, which are given by Equations (8) and (9).

(8) $$Error(R)=\arccos\left[\frac{tr(R_{gt}^{T}R_{pre})-1}{2}\right]$$

(9) $$Error(t)={\left\|t_{gt}-t_{pre}\right\|}_{2}$$

where *R_pre_*, *t_pre_*, *R_gt_*, and *t_gt_* denote the predicted transformation and the true transformation, respectively, and *tr*(*·*) is the trace of the matrix. 

### 5.3. Modelnet40

#### 5.3.1. Unseen Shapes

**Figure 3:**
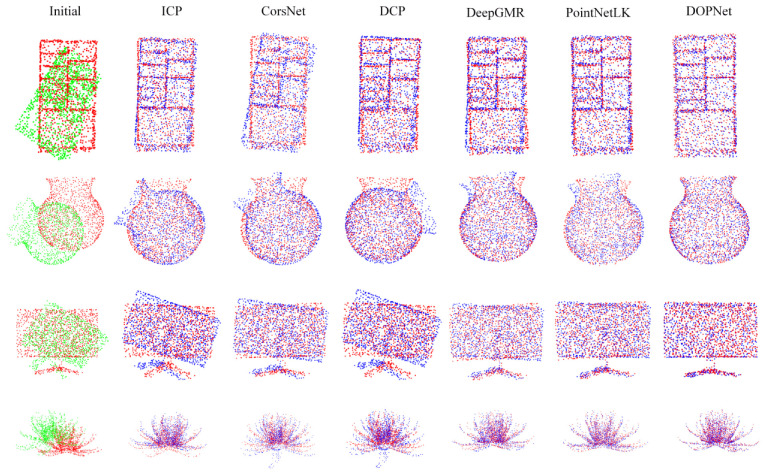
Registration results (red: target point cloud; green: source point cloud; blue: predicted point cloud). The first column shows the initial position of the point clouds, while other columns show the registration results.

In this experiment, ModelNet40 was randomly divided into the training set and the testing set. We used 9843 models as the training set and 2688 models as the testing set. Table 1 shows the results. Our method achieved excellent accuracy, while it was slightly inferior to DeepGMR in translation error. Such slight deficiency might be created by DeepGMR’s ability to capture special position relationships between point sets, while DOPNet learns more comprehensive features. That means that DeepGMR is more sensitive to special geometric structures, which might explain its minor superiority in unseen shapes and inferiority in unseen categories. Figure 3 shows the registration results. It can be seen that the proposed method can still achieve good registration results for highly symmetric structures, repetitive structures, and complex structures, which frequently cause falling into local optima or unsuccessful registration. Furthermore, the flowerpot (one of the special symmetric categories) was well aligned by DOPNet, but the corresponding metrics in AO were still much better than those in TO. Such variation demonstrates that registration errors for these special symmetrical objects cannot be fairly evaluated with the metrics.

#### 5.3.2. Unseen Categories

To evaluate the generalization of these methods, we used the first 20 categories as the training set and the remaining 20 categories as the testing set; note that ICP was evaluated on the testing set. The results are shown in Table 2. The ICP error rate slightly decreased, while the errors of learning-based methods increased due to the differences in features of various categories of point clouds; CorsNet especially almost failed registration. Benefiting from the hybrid features which contain abundant spatial structure information and latent semantic information, DOPNet is less sensitive to the variation of feature styles, it shows the best generalization with the lowest errors.

**Figure 4:**
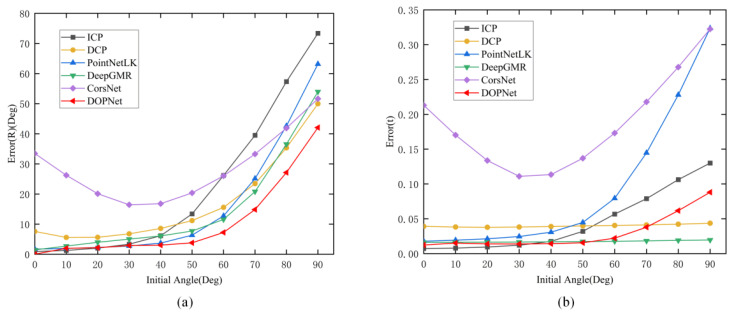
Generalization to the initial rotation angle. (**a**) denotes Error(R), (**b**) denotes Error(t).

Furthermore, in order to evaluate the sensitivity of these methods to the initial rotation angle, ICP and the trained models were tested on the total model data (TO) of the testing set with the range of [0°, 90°] by taking 10° as the step. For brevity, Error(R) and Error(t) were chosen as the bases of evaluation. Figure 4 shows the results. The red folded line denotes DOPNet. DOPNet achieved a similar error rate to other methods in the range of [0°, 50°], and it was slightly inferior in translation error when the rotation angle was bigger. However, on the whole it still showed stronger robustness toward rotation angles. As different types of model data were used in training and testing, the results also further reflect the good generalization of DOPNet. Table 3 and Table 4 show the corresponding quantitative results.

#### 5.3.3. Robustness

Noise is an inevitable disturbance factor affecting registration accuracy. We tested robustness against noise for these methods with the data set and trained models in Section 5.3.1. The noise was sampled from *N*(0, 0.012) and clipped to [−0.5, 0.5]. Table 5 shows the results. As the noise blurred the key features and disturbed the correspondences between *P_S_* and *P_T_*, almost all the metrics of these compared methods became slightly or significantly worse. As similar datasets in Section 5.3.1 were employed to conduct this experiment, DeepGMR still achieved the best performance in translation error for the potential characteristics described in Section 5.3.1. The OA carried out consequential suppression of noise, and our method achieved relatively superior registration results in both TO and AO.

#### 5.3.4. Calculation Efficiency

Calculation efficiency is an important factor in evaluating a registration algorithm. We tested the efficiency of these methods under the same settings as in Section 5.3.1, and the average cost on the TO was used as the time cost of the corresponding method. The results are shown in Table 6. Our method was slightly less efficient than DeepGMR, as DeepGMR has fewer learnable parameters, and it is superior to other registration methods in calculation efficiency. Our registration network was designed in a non-iterative style, and we also took into consideration the latent variation of features during registration in detail. Therefore, DOPNet can not only achieve excellent registration accuracy, it also possesses fairly good calculation efficiency. 

### 5.4. Stanford Point Clouds

**Figure 5:**
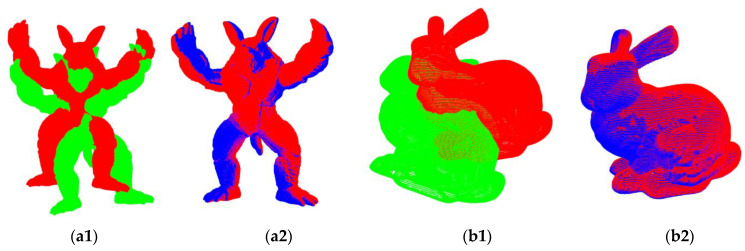
Visual registration results. Red, green, and blue denote the target point cloud, source point cloud, and predicted point cloud, respectively, and (**a1**,**b1**) indicate the initial positions, while (**a2**,**b2**) denote corresponding registration results.

The Stanford 3D Scanning Repository [33] contains many point clouds obtained by scanning real objects. These models are more complex compared with those in Modelnet40. Thus, the Stanford bunny model and armadillo model were utilized to further evaluate the generalization and registration ability of DOPNet. The trained network model and evaluation metrics in Section 5.3.2 were employed to conduct this experiment. Note that 4096 points were sampled from the original models to retain more significant structural information, as the models are much more structurally complicated. The results are shown in Figure 5 and Table 7.

Error(R) increased slightly compared with the corresponding metric of AO in Section 5.3.2, while other metrics improved. Figure 5 shows that DOPNet can still achieve fine registration with this different data set without retraining. The qualitative and quantitative results further prove that DOPNet possesses good generalization and registration.

### 5.5. Ablation Studies

We conducted several ablation experiments on Modelnet40 to dissect DOPNet and understand the value of these modules. All experiments were conducted with the same settings as in Section 5.3.1. The experiments consisted of three parts: replacing DGCNN with PointNet, removing the OA module, and removing the FI module. Error(R) and Error(t) were used as the metrics. The results are shown in Table 8.

The last row denotes DOPNet. It can be seen that the metrics would analogously be slightly worse due to replacing DGCNN with PointNet and removing the FI module. Such variation demonstrates that DGCNN is more capable of extracting representative features than PointNet, and it also verifies the necessity of making features interact between two feature extraction branches for high-quality registration. Not surprisingly, removing OA modules terribly weakens the performance of DOPNet, which establishes that OA modules can effectively extract comprehensive semantic feature information for subsequent procedures. The results support our motivation to construct the network with OA modules.

## 6. Conclusions

We introduced a point cloud registration method based on deep learning, taking into account the calculation efficiency and accuracy of the point cloud registration, and achieving fast and accurate registration. DGCNN was leveraged for embedding point clouds into high-dimensional space, the cascaded OA modules were used for extracting comprehensive features, and the feature interaction module was inserted into the feature extraction pipeline to enhance information connection and registration accuracy. The predicted transformation is given by the MLP. On ModelNet40 and Stanford point cloud datasets, we carried out comprehensive comparison experiments with ICP and some mainstream learning-based methods, while the point clouds were generated by sampling the original models twice independently. The results adequately demonstrate that DOPNet possesses excellent registration ability and efficiency compared with these algorithms. In the future, we will work on solving the network limitations regarding partial-to-partial registration and improving accuracy and applicability based on the idea of predicting overlap masks. 

## Figures and Tables

**Table 1:** Test on unseen shapes.

	Total Objects (TO)				Asymmetrical Objects (AO)			
	Error(R)	RMSE(R)	Error(t)	RMSE(t)	Error(R)	RMSE(R)	Error(t)	RMSE(t)
ICP	9.2680	11.6344	0.0249	0.0283	7.5924	10.6252	0.0213	0.0257
DCP	8.8776	6.5896	0.0386	0.0253	8.2090	6.5152	0.0383	0.0252
PointNetLK	4.1094	6.8172	0.0325	0.0442	2.6626	6.4858	0.0240	0.0408
DeepGMR	6.0436	5.2149	0.0180	0.0119	4.6820	3.8150	0.0180	0.0118
CorsNet	17.6834	11.2690	0.1194	0.0805	17.5577	11.1810	0.1185	0.0793
DOPNet	3.2652	3.0954	0.0202	0.0130	2.3790	3.0954	0.0189	0.0130

**Table 2:** Test on unseen categories.

	TO				AO			
	Error(R)	RMSE(R)	Error(t)	RMSE(t)	Error(R)	RMSE(R)	Error(t)	RMSE(t)
ICP	8.9302	10.5838	0.0236	0.0264	7.7656	10.3687	0.0200	0.0236
DCP	9.7660	6.7074	0.0397	0.0259	8.7916	6.4918	0.0382	0.0245
PointNetLK	4.6391	8.1576	0.0357	0.0523	3.0385	6.3826	0.0265	0.0435
DeepGMR	6.7631	5.8911	0.0171	0.0111	5.6436	4.9185	0.0169	0.0109
CorsNet	18.2673	11.5248	0.1234	0.0825	18.3851	11.6543	0.1259	0.0843
DOPNet	3.4526	3.0481	0.0148	0.0109	2.9152	2.5419	0.0141	0.0101

**Table 3:** Results of Error(R).

Angle	ICP	DCP	PointNetLK	DeepGMR	CorsNet	DOPNet
0	0.9084	7.5824	1.7248	1.4614	33.4483	0.0180
10	1.2587	5.5909	1.9439	2.7263	26.2406	1.9824
20	2.0430	5.6339	2.2575	3.9792	20.1014	2.1643
30	3.3596	6.7853	2.7429	5.0188	16.4299	2.8950
40	6.1502	8.5904	3.7187	6.0896	16.7891	3.0301
50	13.3923	11.1812	6.3421	7.7168	20.3785	3.8056
60	26.1586	15.5790	12.7429	11.5782	26.0397	7.2468
70	39.4913	23.4601	25.1283	20.8181	33.2745	14.8409
80	57.3136	35.3365	42.6074	36.5492	41.8893	27.1315
90	73.3583	49.9891	63.1546	53.9633	51.6397	42.0658

**Table 4:** Results of Error(t).

Angle	ICP	DCP	PointNetLK	DeepGMR	CorsNet	DOPNet
0	0.0074	0.0391	0.0178	0.0161	0.2130	0.0124
10	0.0079	0.0382	0.0193	0.0162	0.1702	0.0152
20	0.0095	0.0378	0.0213	0.0164	0.1337	0.0140
30	0.0121	0.0382	0.0246	0.0166	0.1110	0.0139
40	0.0176	0.0389	0.0308	0.0169	0.1135	0.0143
50	0.0320	0.0398	0.0448	0.0173	0.1370	0.0156
60	0.0567	0.0405	0.0794	0.0177	0.1731	0.0222
70	0.0789	0.0412	0.1447	0.0183	0.2179	0.0379
80	0.1063	0.0423	0.2280	0.0190	0.2678	0.0617
90	0.1300	0.0437	0.3237	0.0196	0.3226	0.0881

**Table 5:** Robustness against noise.

	TO				AO			
	Error(R)	RMSE(R)	Error(t)	RMSE(t)	Error(R)	RMSE(R)	Error(t)	RMSE(t)
ICP	9.6267	11.3117	0.0263	0.0283	8.1309	11.0779	0.0226	0.0253
DCP	9.0785	6.7074	0.0382	0.0251	8.4129	6.6357	0.0301	0.0251
PointNetLK	4.6918	7.3042	0.0375	0.0471	3.0578	6.7635	0.0279	0.0399
DeepGMR	6.0783	5.2392	0.0180	0.0119	4.7300	3.8595	0.0180	0.0118
CorsNet	17.6645	11.2246	0.1199	0.0807	17.7338	11.2575	0.1196	0.0801
DOPNet	3.3255	3.1103	0.0202	0.0131	2.4476	1.9277	0.0189	0.0117

**Table 6:** Calculation efficiency.

Method	ICP	DCP	PointNetLK	DeepGMR	CorsNet	DOPNet
Time (ms)	36.2	114	212	15	80	18

**Table 7:** Quantitative registration results.

Models	Error(R)	RMSE(R)	Error(t)	RMSE(t)
Armadillo	3.3320	1.8380	0.0036	0.0021
Bunny	3.3135	1.8216	0.0031	0.0018

**Table 8:** Ablation studies.

DGCNN	OA	FI	TO		AO	
			Error(R)	Error(t)	Error(R)	Error(t)
	√	√	4.2869	0.0564	3.4803	0.0477
√		√	5.7753	0.0765	5.1483	0.0729
√	√		4.2748	0.0523	3.5402	0.0480
√	√	√	3.2652	0.0202	2.3790	0.0189

## Data Availability

The data presented in this study are openly available in [32,33].
